# An insight into the microphysical attributes of northwest Pacific tropical cyclones

**DOI:** 10.1038/s41598-023-29144-4

**Published:** 2023-03-17

**Authors:** Balaji Kumar Seela, Jayalakshmi Janapati, Pay-Liam Lin, Meng-Tze Lee

**Affiliations:** 1 Department of Atmospheric Sciences, Institute of Atmospheric Physics, National Central University, Zhongli District, Taoyuan City, Taiwan; 2 Earthquake-Disaster and Risk Evaluation and Management Center, National Central University, Zhongli District, Taoyuan City, Taiwan; 3 Research Center for Hazard Mitigation and Prevention, National Central University, Zhongli District, Taoyuan City, Taiwan; 4 Department of Atmospheric and Oceanic Sciences, McGill University, Montreal, QC Canada

**Keywords:** Climate sciences, Hydrology, Natural hazards

## Abstract

Northwestern Pacific (NWP) tropical cyclones (TCs) impose a severe threat to the life and economy of the people living in East Asian countries. The microphysical features, mainly the raindrop size distributions (RSD) of TCs that improve the modeling simulation and rainfall estimation algorithms, are limited to case studies, and an extensive understanding of TCs’ RSD is still scarce over the northwest Pacific. Here, we examine a comprehensive outlook on disparities in microphysical attributes of NWP TCs with radial distance and storm type, using sixteen years of disdrometer, ground-based radar, and reanalysis datasets in north Taiwan. We find that dominant stratiform precipitation in the inner rainbands leads to the occurrence of more bigger drops in the inner rainbands than the inner core and outer rainbands. Moreover, a decrease in mass-weighted mean diameter and rainfall rate with radial distance is associated with a reduction in moisture availability for various circumstances, and this association is deceptive in intense storms. Our findings give an insight into crucial processes governing microphysical inequalities in different regions of NWP TCs, with implications for the ground-based and remote-sensing rainfall estimation algorithms.

## Introduction

The Northwestern Pacific (NWP) Tropical cyclones (TCs) (also called typhoons) associated with torrential rainfall attribute a severe threat to the life and economy of people living in East Asian countries^1–3^, and are accountable for floods and earth surface processes^4–7^, which emphasizes the significance of acquiring an enhanced understanding of rain and cloud microphysics of TCs for the accurate prediction of rainfall and intensity^8^. The precipitation microphysics of TCs, especially the raindrop size distribution (RSD), has been a paramount consideration in advancing the radar rainfall estimation algorithms and microphysical parameterization^9–12^.

Owing to the significant contribution of TC RSDs in hydrometeorology and earth surfaces process, there has been increasing interest in exploring the RSD information of TCs from different oceanic regions^11,13–17^. Conversely, most previous observations were conducted with RSD samples using a limited number of TCs or a portion of TC rainbands. Studies performed with numerical simulations and remote sensing data demonstrated the disparities in convection and precipitation distribution with radial distance from the TC center^18–24^. In addition, despite the limited number of TCs measurements, recent studies have hinted at the inequalities in TC’s RSD with radial distance^16,17^. Nonetheless, up to now, an extensive framework that emphasizes the RSD features of TCs with their radial distance and intensity is yet to be known.

Therefore, it is imperative to study the RSD of TCs in a long-term perspective to deduce their robust characteristics at different radial distances and category types. In this work, using long-term ground-based (disdrometer and radar) and re-analysis data sets, we elucidate the microphysical processes liable for the RSD changes with TCs radial distance and intensity. The results depict a decrease in rainfall rate and mass-weighted mean diameter with radial distance from the TC center, with a substantial decreasing pattern for intense TCs (CAT15) than the tropical storm category TCs. The microphysical attributions and rainfall retrieval relations disclosed for different intensities and radial distances can improve the TCs modeling simulations and radar precipitation estimation algorithms. The results present in this study provide conceivable microphysical attributes responsible for the RSDs variations at different radial distances and offer possible implications for the rain retrieval algorithms of ground-based and remote sensing radars.

## Results

### Radial variation of RSD parameters and mean reflectivity profiles

**Figure 1:**
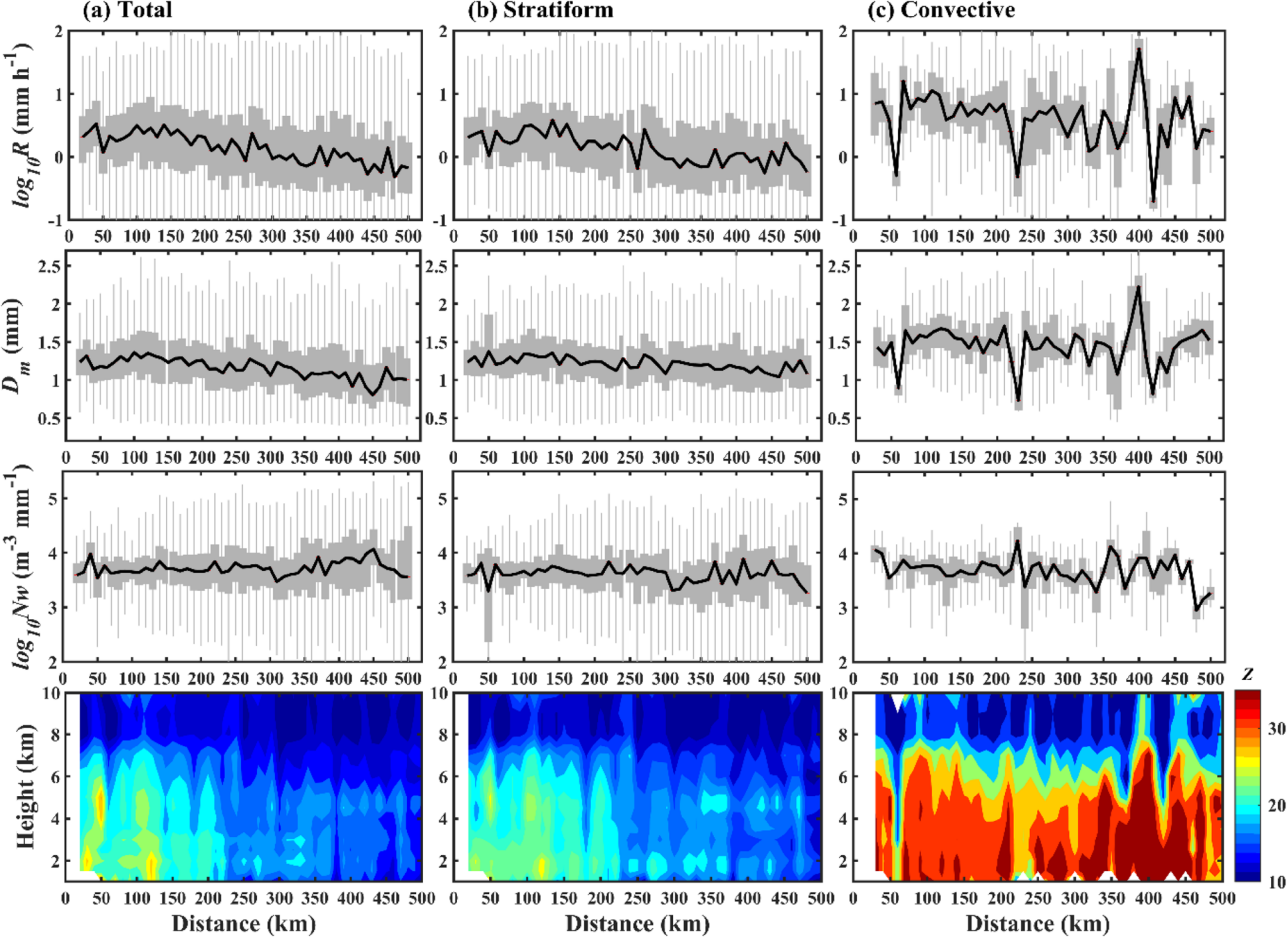
Variations in RSD parameters with radial distance. Distribution of rainfall rate (log_10_*R*, *R* is in mm h^–1^) (first row), mass-weighted mean diameter (*D*_*m*_, mm) (second row), normalized intercept parameter (log_10_*N*_*w*_, *N*_*w*_ is in m^–3^ mm^–1^) (third row), and vertical profile of mean radar reflectivity (Z, dBZ) (fourth row) with radial distance (every 10 km) for all category TCs’ (**a**) total, (**b**) stratiform, (**c**) convective rainfall. RSD parameters in first three rows are displayed with box plots. The gray areas in first three rows denote the 25th–75th percentile of the RSD parameters, and the black solid lines denoted the median values at every 10 km.

Figure 1 displays the distribution of rainfall rate (log_10_*R*, *R* is in mm h^–1^), mass-weighted mean diameter (*D*_*m*_, mm), normalized intercept parameter (log_10_*N*_*w*_, *N*_*w*_ is in m^–3^ mm^–1^), and mean radar reflectivity (*Z*, dBZ) profiles at 10 km radial distances from TC center. For the total precipitation of all TCs (Fig. 1a), rainfall rate and *D*_*m*_ values gradually increase from 50 to 100 km and then decrease up to 500 km. The normalized intercept parameter (log_10_*N*_*w*_) gradually increases while moving radially outward from TC center. The contour plot of mean *Z* profiles for every 10 km shows higher *Z* values within 50 km, 100–130 km, and around 200 km, and gradually decrease beyond 200 km from the TC center. In the cold rain regions of TCs (above 0 °C isotherm height, i.e., ~ 5 km), the mean reflectivity values greater than 25 dBZ are visible around 50 km from the TC center. However, in the warm rain region of TCs, mean *Z* values greater than 25 dBZ are apparent around 50 km, 130 km, and 200 km. A further classification of all TCs precipitation into stratiform and convective types shows that the RSD parameters (rainfall rate, *D*_*m*_, and log_10_*N*_*w*_), and mean *Z* profiles in stratiform rain follow a similar distribution pattern to that of the total rainfall of all TCs. In contrast to stratiform precipitation, the RSD parameters in convective precipitation show a sharp decrease from TC center to 50 km, and these parameters increase above 200 km from the TC center. In the warm rain region of all TCs convective precipitation, the mean *Z* profiles show higher values (> 35 dBZ) up to 100 km, and above 200 km from the TC center. The extent of deep convective cores with *Z* values greater than 35 dBZ into the cold rain region is more predominant, especially from 340 to 450 km from the TC center.

### Radial variation of RSD parameters and reflectivity profiles with TC category

**Figure 2:**
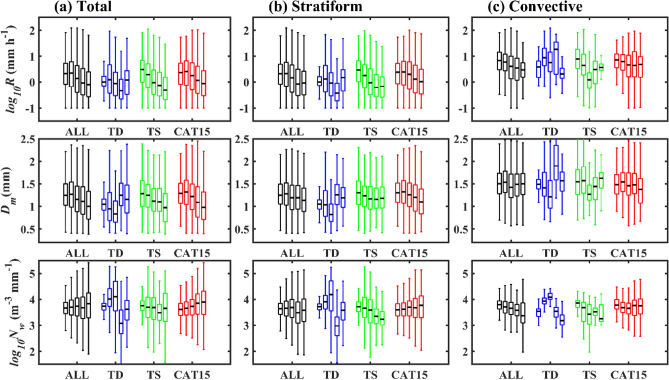
Variations in RSD parameters with radial distance for TCs of different category. Distribution of rainfall rate (log_10_*R*, *R* is in mm h^–1^) (first row), mass-weighted mean diameter (*D*_*m*_, mm) (second row), and normalized intercept parameter (log_10_*N*_*w*_, *N*_*w*_ is in m^–3^ mm^–1^) (third row) for (**a**) total, (**b**) stratiform, and (**c**) convective precipitations of all and different category (TD, TS, and CAT15) TCs at different radial distances [radial distance 1 (RD1: 0–100 km), radial distance 2 (RD2: 100–200 km), radial distance 3 (RD3: 200–300 km), radial distance 4 (RD4: 300–400 km), radial distance 5 (RD5: 400–500 km)]. Five box plots in each TCs category (All, TDs, TSs, and CAT15) denote RD1 to RD5, respectively, from left to right.

Observed NWP TCs are segregated to tropical depressions (TDs), tropical storms (TSs), and category 1–5 (CAT15) with radial distances of 100 km each from the TC center ((RD1: 0–100 km, RD2: 100–200 km, RD3: 200–300 km, RD4: 300–400 km, RD5: 400–500 km; supplementary Fig. 3). Figure 2 displays the distribution of RSD parameters (log_10_*R*, *D*_*m*_, and log_10_*N*_*w*_) for total, stratiform and convective precipitations of different category TCs. For all TCs (Fig. 2a), a slight increase (from RD1 to RD2) and a further decrease (from RD2 to RD5) in median values of *D*_*m*_ and log_10_*R* with radial distance is apparent, which can be attributed to the relatively more significant number of small drops in outer regions (RD3–RD5) than inner regions (RD1 and RD2) (Supplementary Fig. 4). For TCs of all categories, the median values of log_10_*N*_*w*_ increase with the increase in radial distance. A further classification of all TCs into three categories (TDs, TSs, and CAT15) depicts that the RSD parameters (*D*_*m*_, log_10_*N*_*w*_, and log_10_*R*) of TDs have an inhomogeneous distribution with radial distance. However, for TSs, median values of *D*_*m*_ and log_10_*R* decrease and log10*N*_*w*_ increase, except at RD4. In the case of TCs of CAT15, *D*_*m*_ and log_10_*R* median values increase (RD1–RD2) and then decrease, and log_10_*N*_w_ values increase. Segregation of TCs of different categories into two precipitation (stratiform and convective) types shows that the RSD parameters (log_10_*R*, *D*_*m*_, and log_10_*N*_*w*_) in stratiform precipitation (Fig. 2b) follow the similar tendency to that of the total precipitation (Fig. 2a). In convective precipitation, TSs’ RSD parameters (log_10_*R* and* D*_*m*_) decrease (RD1–RD3) and then increase (RD3–RD5). Whereas, for TCs of CAT15, the median values of the three RSD parameters decrease from the TC center. Despite that, for the first two radial distances (RD1–RD2), TCs of CAT15 show higher (lower) median values of *D*_*m*_ (log_*10*_*N*_w_) than TDs and TSs, and an opposite feature appears for the last two radial distances (RD4 and RD5) (Supplementary Fig. 5). Nevertheless, in most of the radial distances, rainfall rates are higher in TCs of CAT15 than TSs (except at RG1) (Supplementary Fig. 5), which can be attributed to a relatively more number of small size drops (smaller *D*_*m*_ and larger log_10_*N*_*w*_ values) in TSs than CAT15.

**Figure 3:**
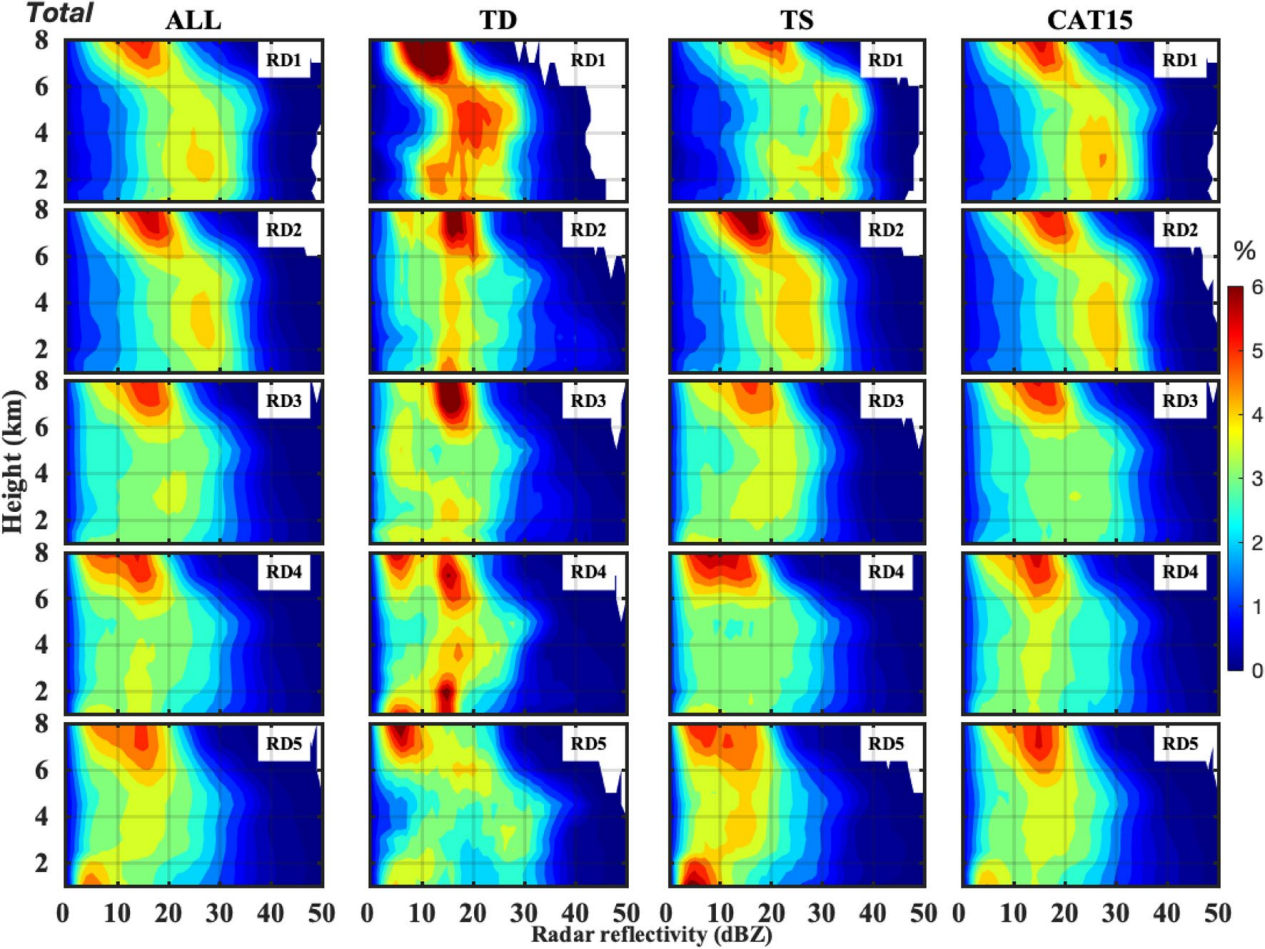
Echo profiles of different category TCs at different radial distances for total precipitation. The contour frequency by altitude diagrams (CFADs) of radar reflectivity for all (first column), TDs (second column), TSs (third column) and CAT15 (fourth column) TCs for different radial distances (RD1: 0–100 km, RD2: 100–200 km, RD3: 200–300 km, RD4: 300–400 km, and RD5: 400–500 km).

Figure 3 demonstrates the contour frequency by altitude diagrams (CFADs) of radar reflectivity for different radial distances (RD1–RD5) of TCs. For all TCs, the occurrence of 30 dBZ reflectivity is extended up to 7 km in RD1 and RD2, and its extent is limited up to around 6 km for the rest of the radial distances (RD3, RD4, and RD5). Below the melting layer (< 5 km), the reflectivity occurrence frequency of 2–4% is spread between 10–36 and 10–38 dBZ, respectively, for RD1 and RD2; nonetheless, RD2 exhibit relatively higher percentage (> 3.5%) than RD1. On the other hand, in the warm rain region (< 5 km), the reflectivity occurrence frequency of 2–4% shifts towards lower reflectivity values from RD3 to RD5 (~ 2 to 35 dBZ, 2–32 dBZ, and 2–30 dBZ for RD3, RD4, and RD5, respectively). The radar reflectivity higher percentage occurrence (> 4%) ranges between 15–35 dBZ, 25–35 dBZ, 18–28 dBZ, 2–18 dBZ, and 2–15 dBZ, respectively, for RD1, RD2, RD3, RD4, and RD5. The radar reflectivity CFADs of TDs exhibit an irregular distribution among five radial distances (RD1–RD5) with no consistent behavior from RD1 to RD5. Whereas, in the warm rain region (< 5 km) of TSs and CAT15, radar reflectivity occurrence frequency exhibit nearly vertical structure in the first two radial distances (RD1 & RD2) with a greater percentage occurrence in RD2 (RD1) for CAT15 (TSs). The reflectivity occurrence frequency shows a gradual downward decrease in the warm rain region of TSs and CAT15 from RD3 to RD5; nonetheless, for a given region (except RD1), TCs of CAT15 show a higher reflectivity occurrence frequency than TSs.

**Figure 4:**
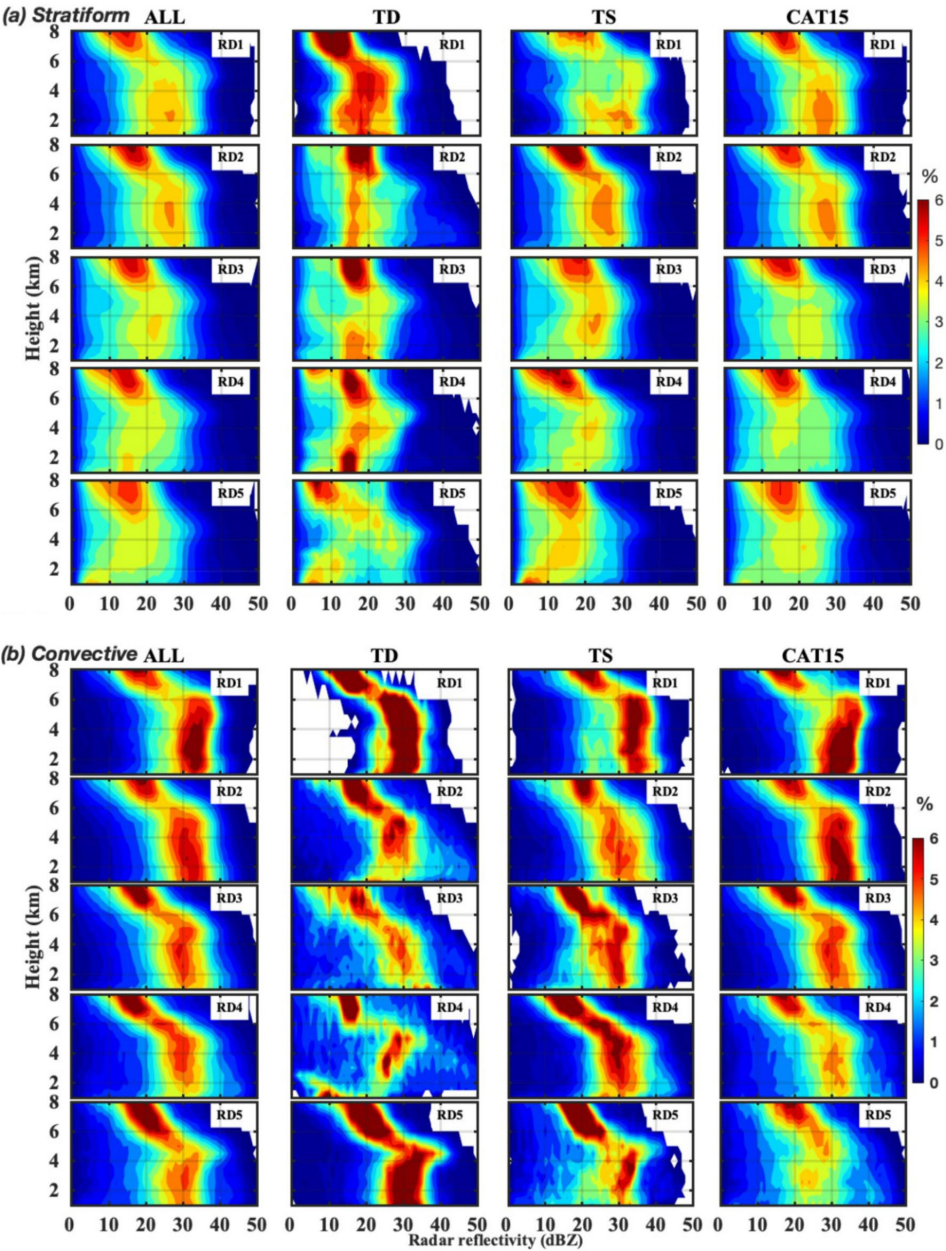
Echo profiles of different category TCs at different radial distances for stratiform and convective precipitation. Same as Fig. 3 but for (**a**) stratiform and (**b**) convective precipitation.

The stratiform precipitation CFADs (Fig. 4a) of different category TCs exhibit nearly identical characteristics to the total rainfall. In the warm rain region of TS, the reflectivity occurrence of 4–5% spread decreases (~ 20–35 dBZ, 12–30 dBZ, 10–25 dBZ, 10–25 dBZ, 5–20 dBZ, for RD1, RD2, RD3, RD4, and RD5, respectively) radially outwards from the TC center. Despite the similar tendency of CAT15 stratiform CFADs to that of the TSs, the warm rain region of CAT15 shows a slightly higher occurrence percentage in RD2 than in RD1. The convective precipitation of all TCs show higher CFAD occurrence in RD1, and its frequency decrease to lower reflectivity values with the radial distance from the TCs’ centers (Fig. 4b). The convective precipitation CFAD distribution patterns of TSs and CAT15 are nearly identical to that of the stratiform precipitation with relatively higher occurrence percentage. Similar to the TDs’ total rainfall, stratiform and convective precipitations also exhibit inconsistent tendencies with the increase in the radial distance.

### Distributions of RSD parameters and reflectivity profiles in different rain regions

**Figure 5:**
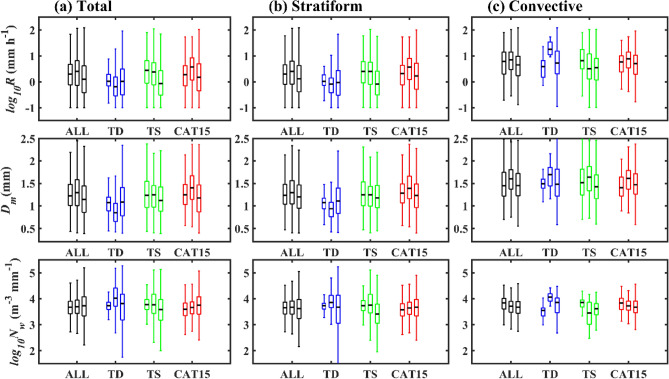
Variations in RSD parameters in different rain regions (IC: inner core, IB: inner rainbands, and OB: outer rainbands) of TCs. Distribution of rainfall rate (log_10_*R*, *R* is in mm h^–1^) ( first row), mass-weighted mean diameter (*D*_*m*_, mm) (second row), normalized intercept parameter (log_10_*N*_*w*_, *N*_*w*_ is in m^–3^ mm^–1^) (third row) for (**a**) total (**b**) stratiform (**c**) convective precipitation for TCs of all category, TDs, TSs, and CAT15. Three boxplots in each TCs category denote the IC, IB and OB, respectively from left to right.

**Figure 6:**
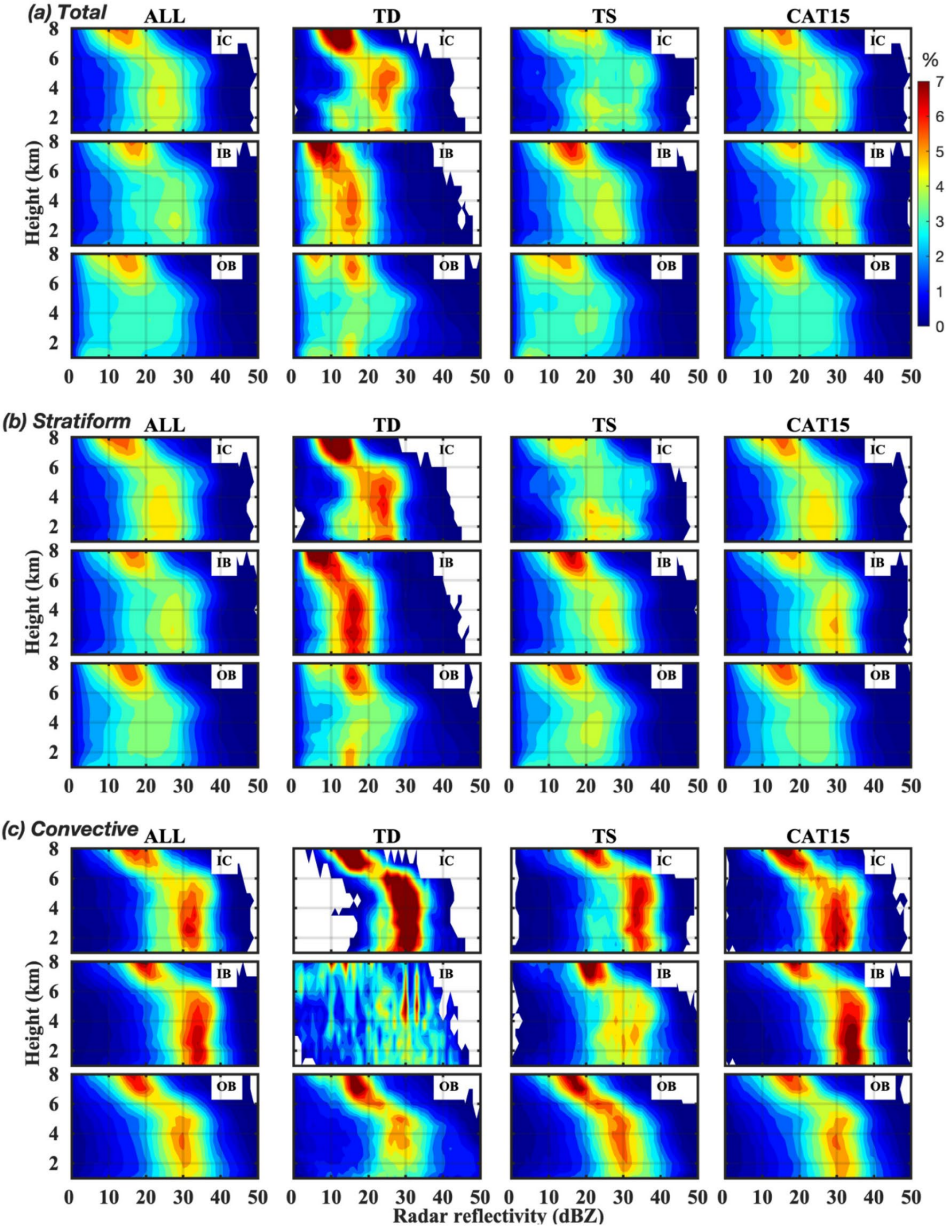
Echo profiles of different category TCs at different rain regions. The contour frequency by altitude diagrams (CFADs) of radar reflectivity for TCs of all categories, TDs, TSs and CAT15 at different rain regions (IC: inner core, IB: inner band, OB: outer bands). (**a**) Total precipitation, (**b**) stratiform precipitation, and (**c**) convective precipitation.

To further investigate the disparities in the RSDs of TCs in different rain regions (Inner core: IC, Inner rainbands: IB, and outer rainbands: OB), the distribution of the RSD parameters at three rain regions of WP TCs are illustrated in Fig. 5. The total precipitation of all, and CAT15 TCs show relatively higher *D*_*m*_ and rainfall rate values in IB than IC and OB. On the other hand, TDs show smaller *D*_*m*_ and rainfall rate values in IB than rest two regions. The normalized intercept parameter show an increase from IC to OB in all and CAT15 TCs; however, they decrease (increase and then decrease) in TSs (TDs). In the case of stratiform precipitation(Fig. 5b), TCs of all categories and CAT15 show a larger log_10_*N*_*w*_ (*D*_*m*_ and rainfall rate) value in the OB (IB). On the other hand, the RSD parameters show decreasing (increasing and then decreasing) tendency in TSs (TDs) from IC to OB. In the case of convective precipitation (Fig. 5c), TCs of all categories and CAT15 show higher *D*_*m*_ and log_10_*R* (log_10_*N*_*w*_) values in the IB (IC) region. However, for TDs, three RSD parameters increase and then decrease from IC to OB. Figure 6 illustrates the WP TCs CFADs in different rain regions (IC, IB, and OB). Among three rain regions of ALL, TD, and CAT15 TCs, OB show weaker CFADs than the IC and IB. For ALL TCs, the extent of 30 dBZ reflectivity occurrence percentage (~ 4 to 5%) is relatively higher (~ 6 km) in the IC than in the IB and OB regions (< 6 km). However, in the warm rain region of all TCs, the reflectivity occurrence of 4–5% is extended between 20–30 dBZ in IC and 22–32 dBZ in the IB. In the case of TDs, the extent of 20–30 dBZ reflectivity occurrence percentage (~ 5–7%) is relatively higher in the IC, and this occurrence percentage is shifted towards the lower reflectivity values (10–20 dBZ) in the IB regions. In the warm rain region of TSs, a 3–4% frequency spread of radar reflectivity moves to lower reflectivity values from IC to OB (20–35 dBZ, 10–30 dBZ, and 5–20 dBZ in IC, IB, and OB, respectively). In the warm rain region of intense TCs (CAT15), 4–5% radar reflectivity occurrence spreads from ~ 20–30 dBZ in the IC, and 25–35 dBZ in the IB regions. In the stratiform and convective regimes of all TCs, despite the differences in the occurrence of stratiform (~ 4–5%) and convective (~ 5–6%) rainfall, the extent of 20–30 dBZ reflectivity is relatively higher in the IC (~ 6 km) than IB (< 6 km) region. Similar characteristics can be seen for TDs and TSs. On the contrary, stratiform rainfall of intense TCs shows a relatively deeper and broader spread of 4–5% echo in the IB region (~ 6 km and 25–35 dBZ) than in the IC region (< 6 km and 20–30 dBZ). Similarly, the convective rainfall of intense TCs also exhibit relatively deeper and wider spread of 5–6% echo in the IB region (~ 6 km and 30–38 dBZ) than the IC region (< 6 km and 25–35 dBZ).

## Discussion

**Figure 7:**
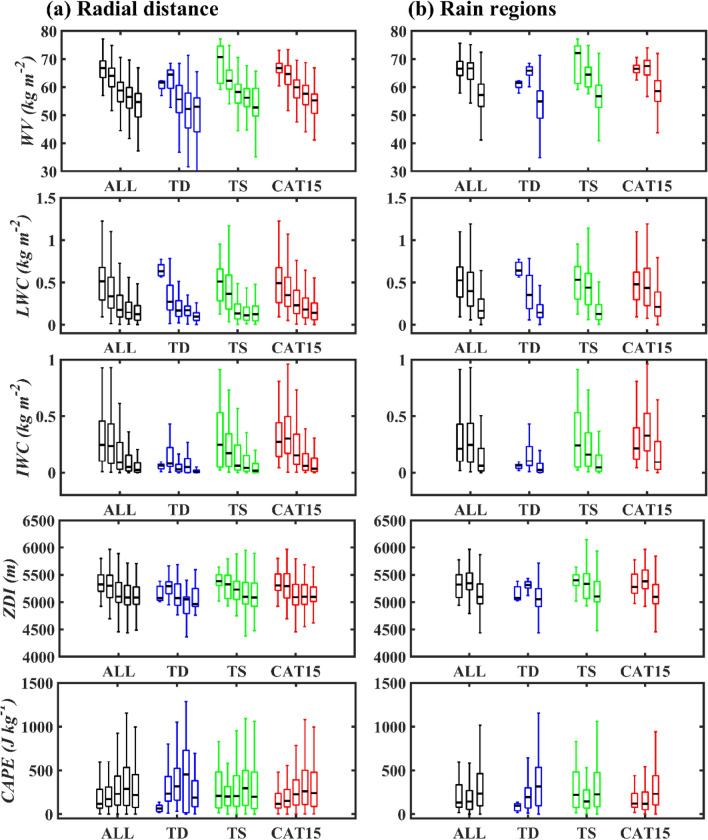
Cloud and precipitation parameters of TCs. Distribution of water vapor (*WV*, Kg m^–2^), total column liquid water content (*LWC,* Kg m^–2^), total column ice water content (*IWC*, Kg m^–2^), zero degree isotherm (*ZDI*, m), and convective available potential energy (*CAPE*, J Kg^–1^) for All, TD, TS, and CAT15 TCs at (**a**) different radial distances (RD1 to RD5) and (**b**) rain regions (IC, IB, and OB). Five box plots (from left to right) for each category TCs in (**a**) corresponds to RD1 to RD5. Three box plots (from left to right) for each category TCs in (**b**) corresponds to IC, IB, and OB.

The opportunity to interpret the plausible inconsistencies in the raindrop size distribution (RSD) characteristics of NWP TCs at different radial distances (RD1–RD5) and rain regions (IC, IB, and OB) can be apprehended with the changes in thermodynamics and microphysics of precipitation. These changes can be related to crucial parameters like radar reflectivity profiles, moisture availability, melting layer height, and total column liquid/ice water content. Distributions of these cloud and precipitation parameters (total column water vapor, liquid water content, ice water content, zero degrees isotherm, convective available potential energy) at different radial distances (RD1–RD5) and rain regions (IC, IB, and OB) are portrayed in Fig. 7. An apparent decrease in total column water vapor and liquid water content with the increase in radial distance from TC center can be seen for the TCs of all categories, with a much more clear tendency in intense TCs (CAT15) (Fig. 7a). However, the zero-degree isotherm and the total column ice water content values increase from RD1 to RD2 and then decrease from RD2 to RD5. Similarly, total column water vapor and liquid water content show relatively higher values in IC and IB than OB regions. The Zero-degree isotherm and total column ice water content show higher values in IB than the rest two regions with pronounced characteristics in TCs of CAT15 (Fig. 7b). The decreasing tendency in total column water vapor and liquid water content can be attributed to the decrease in rainfall amounts with the radial distance from TCs’ center, especially for TSs and CAT15 TCs (as shown in Fig. 1). As the increase in small drops and decrease in large drops can be ascribed to the reduction in *D*_*m*_ values, radial decrease (increase) in *D*_*m*_ (log_10_*N*_*w*_) values signifies the increase in small drops leading to reduction in the rainfall amount from TC center to outer regions, i.e., from RD1 to RD5 (Fig. 1).

Because of the adaptation of present TCs’ rain region segregation into IC, IB, and OB using Yang et al.^20^, the RD1 (0–100 km), RD2 (100–200 km), and RD3–RD5 (200–500 km) can be approximated to the inner core, inner rainbands, and outer rainbands. In the cold regions (above the melting layer) of CAT15 TCs, greater reflectivity values in the inner core suggest that the inner core is characterized by strong convective activity than inner rainbands, and this intense convection can radially transfer the small ice particle in the higher altitudes of the inner core to inner rainbands^22^. The small ice particles radially transferred from inner core to inner rainbands by deep and intense convection can suppress the vertical extent of convection and enhance robust stratiform precipitation in the inner rainbands. Further, relatively higher zero-degree isotherms (proxy to the melting layer height, Fig. 7) indicate the dominance of strong stratiform signatures in the inner rainbands than in the inner core. Relatively higher melting layer heights in the inner rainbands provide sufficient time for the growth of ice crystals to large sizes via aggregation and vapor deposition, and these large ice particles melt entirely once they cross the melting layer resulting in large raindrops. In TCs of CAT15, dominated breakup processes caused by the intense convection in the inner core (or RD1) and relatively deeper stratiform precipitations in the inner rainbands (or RD2) lead to relatively large size drops in inner rainbands (or RD2). Besides, fully-grown raindrops under intense convection in the inner core (or RD1) are smaller than the stratiform rainfall in the inner rainbands (or RD2), resulting in relatively higher *D*_*m*_ values in inner bands than in the inner core^25,26^. Parallel to the findings of the present study, Bao et al.^17^ also reported similar RSD characteristics at different radial distances/rain regions of typhoon Lekima (2019) measured over Eastern China. Moreover, the RSD features documented for different rain regions of southern Ocean TCs are likewise to that of the present study^16^. Variability in RSD parameters with radial distance gives a clue to estimate the region-specific rainfall estimation relations such as radar reflectivity–rainfall rate (*Z*–*R*), shape–slope (*μ*–*Λ),* and mass-weighted mean diameter–rainfall rate (*D*_*m*_–*R*) relations. The *Z*–*R*, *μ*–*Λ,* and *D*_*m*_–*R* relations estimated for NWP TCs of different intensity and radial distances are provided in Table 1. A remarkable distinction in the estimated RSD relations for different radial distances and rain regions of TCs of the different categories suggests the importance of adopting radial distance/rain region-specific empirical relations while evaluating rainfall retrievals for the ground-based and remote sensing radars. In estimating the rainfall amounts from the radar reflectivity measurements of the ground-based radars, the Taiwan QPESUMS (Quantitative Precipitation Estimation (QPE) and Segregation Using Multiple Sensors) system adopts either precipitation-specific *Z*–*R* relations (*Z* = 300*R*^1.4^ for convective and *Z* = 32.5*R*^1.65^ for stratiform precipitation) or elsewhere relations (Z = 32.5*R*^1.65^/Z = 300*R*^1.25^)^27–29^. It is important to note that the *Z*–*R* relations adopted in Taiwan QPESUMS are quite different from the *Z*–*R* relations estimated in this study. Moreover, even though there were reports on the Z–*R* relations of NWP TCs for the Taiwan region, such estimates were constrained to the RSD samples of limited TCs^11^. Hence, the *Z*–*R* relations estimated in this paper could offer better QPE results for Taiwan typhoon rainfall events. The slope-shaper relations established for different rain regions of TCs of different intensity categories could advance the TCs’ RSD estimates for the ground-based polarimetric radars^30^. Furthermore, shape-slope and *R*–*D*_*m*_ relations derived in this study can progress the precipitation estimation algorithm of remote-sensing instrumentation (Global precipitation measurement mission- dual-frequency precipitation radar : GPM DPR)^31^.

**Table 1:** Rainfall estimation relations.

TC category	Radial distances/rain regions	Empirical relations		
		*Z*–*R*	*μ*–*Λ*	*D*_*m*_–*R*
ALL	RD1	*Z* = 211.85* R*^1.35^	*μ* = *− 0.05 Λ*^*2*^ + *2.10 Λ-4.53*	*D*_*m*_ = 1.12 *R*^0.15^
	RD2	*Z* = 205.55 *R*^1.35^	*μ* = *− 0.06 Λ*^*2*^ + *2.21 Λ-4.65*	*D*_*m*_ = 1.11 *R*^0.16^
	RD3	*Z* = 199.87 *R*^1.34^	*μ* = *− 0.04 Λ*^*2*^ + *1.76 Λ-2.43*	*D*_*m*_ = 1.10 *R*^0.15^
	RD4	*Z* = 202.54 *R*^1.36^	*μ* = *− 0.04 Λ*^*2*^ + *1.68 Λ-2.04*	*D*_*m*_ = 1.11 *R*^0.16^
	RD5	*Z* = 174.21 *R*^1.39^	*μ* = *− 0.03 Λ*^*2*^ + *1.53 Λ-1.53*	*D*_*m*_ = 1.05 *R*^0.17^
	IC	*Z* = 214.89* R*^1.34^	*μ* = *− 0.04 Λ*^*2*^ + *1.85 Λ-3.63*	*D*_*m*_ = 1.12 *R*^0.15^
	IB	*Z* = 201.79 *R*^1.38^	*μ* = *− 0.06 Λ*^*2*^ + *2.37 Λ-5.32*	*D*_*m*_ = 1.11 *R*^0.16^
	OB	*Z* = 196 *R*^1.36^	*μ* = *− 0.04 Λ*^*2*^ + *1.78 Λ-2.61*	*D*_*m*_ = 1.09 *R*^0.16^
TD	RD1	*Z* = 173.58 *R*^1.57^	*μ* = *− 0.18 Λ*^*2*^ + *3.81 Λ-10.19*	*D*_*m*_ = 1.01 *R*^0.22^
	RD2	*Z* = 136.75 *R*^1.45^	*μ* = *− 0.04 Λ*^*2*^ + *1.74 Λ-2.53*	*D*_*m*_ = 0.91 *R*^0.21^
	RD3	*Z* = 130.61 *R*^1.28^	*μ* = *− 0.00 Λ*^*2*^ + *0.76 Λ* + *2.53*	*D*_*m*_ = 0.93 *R*^0.17^
	RD4	*Z* = 319.91 *R*^1.26^	*μ* = *− 0.05 Λ*^*2*^ + *1.76 Λ-1.85*	*D*_*m*_ = 1.34 *R*^0.11^
	RD5	*Z* = 217.07 *R*^1.43^	*μ* = *− 0.04 Λ*^*2*^ + *1.79 Λ-2.96*	*D*_*m*_ = 1.14 *R*^0.17^
	IC	*Z* = 171.12 *R*^1.59^	*μ* = *− 0.18 Λ*^*2*^ + *3.81 Λ-10.19*	*D*_*m*_ = 1.01 *R*^0.22^
	IB	*Z* = 133.77 *R*^1.42^	*μ* = *− 0.03 Λ*^*2*^ + *1.67 Λ-3.97*	*D*_*m*_ = 0.9 *R*^0.18^
	OB	*Z* = 189.29 *R*^1.32^	*μ* = *− 0.03 Λ*^*2*^ + *1.44 Λ-0.76*	*D*_*m*_ = 1.08 *R*^0.16^
TS	RD1	*Z* = 173.52 *R*^1.45^	*μ* = *− 0.05 Λ*^*2*^ + *2.13 Λ-4.24*	*D*_*m*_ = 1.03 *R*^0.19^
	RD2	*Z* = 209.44 *R*^1.31^	*μ* = *− 0.04 Λ*^*2*^ + *1.77 Λ-2.44*	*D*_*m*_ = 1.13 *R*^0.14^
	RD3	*Z* = 207.79 *R*^1.27^	*μ* = *− 0.04 Λ*^*2*^ + *1.8 Λ-2.78*	*D*_*m*_ = 1.14 *R*^0.12^
	RD4	*Z* = 239.18 *R*^1.37^	*μ* = *− 0.04 Λ*^*2*^ + *1.76 Λ-2.42*	*D*_*m*_ = 1.18 *R*^0.17^
	RD5	*Z* = 184.50 *R*^1.43^	*μ* = *− 0.03 Λ*^*2*^ + *0.48 Λ-0.81*	*D*_*m*_ = 1.08 *R*^0.18^
	IC	*Z* = 168.19 *R*^1.45^	*μ* = *− 0.05 Λ*^*2*^ + *2.12 Λ-4.08*	*D*_*m*_ = 1.02 *R*^0.19^
	IB	*Z* = 190.39 *R*^1.33^	*μ* = *− 0.02 Λ*^*2*^ + *1.45 Λ-1.59*	*D*_*m*_ = 1.08 *R*^0.15^
	OB	*Z* = 218.59 *R*^1.33^	*μ* = *− 0.05 Λ*^*2*^ + *2.01 Λ-3.22*	*D*_*m*_ = 1.16 *R*^0.14^
CAT15	RD1	*Z* = 238.98 *R*^1.29^	*μ* = *− 0.05 Λ*^*2*^ + *2.12 Λ-4.89*	*D*_*m*_ = 1.18 *R*^0.12^
	RD2	*Z* = 221.03 *R*^1.34^	*μ* = *− 0.06 Λ*^*2*^ + *2.38 Λ-5.68*	*D*_*m*_ = 1.14 *R*^0.15^
	RD3	*Z* = 205.29 *R*^1.36^	*μ* = *− 0.04 Λ*^*2*^ + *1.83 Λ-2.70*	*D*_*m*_ = 1.11 *R*^0.16^
	RD4	*Z* = 174.90 *R*^1.40^	*μ* = *− 0.04Λ*^*2*^ + *1.64 Λ-1.92*	*D*_*m*_ = 1.04 *R*^0.18^
	RD5	*Z* = 163.88 *R*^1.37^	*μ* = *− 0.02 Λ*^*2*^ + *1.44 Λ-1.20*	*D*_*m*_ = 1.01 *R*^0.17^
	IC	*Z* = 239.2 *R*^1.29^	*μ* = *− 0.04 Λ*^*2*^ + *1.85 Λ-4.04*	*D*_*m*_ = 1.18 *R*^0.12^
	IB	*Z* = 233.86 *R*^1.32^	*μ* = *− 0.07 Λ*^*2*^ + *2.54 Λ-6.18*	*D*_*m*_ = 1.18 *R*^0.14^
	OB	*Z* = 190.6 *R*^1.38^	*μ* = *− 0.04 Λ*^*2*^ + *1.84Λ-2.97*	*D*_*m*_ = 1.07 *R*^0.17^

Radar reflectivity–rainfall rate (*Z*–*R*), shape–slope (*μ*–*Λ*), and mass-weighted mean diameter–rainfall rate (*D*_*m*_–*R*) relations for different radial distances and rain regions of NWP TC.

## Methods

### Data

The tropical cyclones (TCs) track information (for the years 2005–2020) is obtained from the best track archive of the U.S. Navy’s Joint Typhoon Warning Center (JTWC: http://www.usno.navy.mil/NOOC/nmfc-ph/RSS/jtwc/best_tracks/), which provides TC’s center, longitude, latitude, maximum sustained surface wind speed, and minimum central pressures (Supplementary Fig. 1a). Based on Saffir-Simpson Scale, the TCs considered in this study are classified as a tropical depression (TD), tropical storm (TS), and category 1–5 (CAT15), respectively, if the maximum surface wind speed near the center of TCs is < 34 knots, 34–63 knots and ≥ 64 knots.

The raindrop size distribution (RSD) measurements from the Joss‐Waldvogel Disdrometer (with 1-min sampling interval)^32^ installed at National Central University, Taiwan (24° 58′ N, 121° 10′ E), are used for the TCs rainy periods during 2005–2020 (Supplementary Fig. 1b). The raw spectra of the disdrometer measurements are used to estimate the raindrop concentration (*N(D)*, mm^–1^ m^–3^), rainfall rate (*R*, mm h^–1^), radar reflectivity factor (*Z*, mm^6^ m^–3^), liquid water content (*W*, g m^–3^), mass-weighted mean diameter (*D*_*m*,_ mm), normalized intercept parameter (*N*_*w*_, m^–3^ mm^–1^)*,* shape parameter (*μ,*-), slope parameter *(Λ*, mm^–1^)^33–35^. The 1-min RSD samples with rainfall rates greater than 0.1 mm h^–1^ are considered in the present study. A good agreement between the disdrometer measured daily accumulated rainfall amounts and the collocated rain gauge for the considered TCs gives the trustworthiness of the disdrometer measurements for further analysis (Supplementary Fig. 2). One-min RSD samples with rainfall rate great than 5 mm h^−1^, *μ* and *Λ* values less than 20 and 20 mm^−1^, respectively, are used to estimate the *μ*–*Λ* relations^36^.

The RSD measurements of the disdrometer are considered as TC’s RSDs if the distance between the disdrometer site and the TC center is ≤ 500 km. Furthermore, based on the distance between the disdrometer site and TC center, the rain regions of the NWP TCs are classified into five radial distances, i.e., radial distance 1 (RD1), radial distance 2 (RD2), radial distance 3 (RD3), radial distance 4 (RD4) and radial distance 5 (RD5), if the distances between the TC center and disdrometer site are 0–100 km, 100–200 km, 200–300 km, 300–400 km, and 400–500 km, respectively (Supplementary Fig. 3). Furthermore, the rain regions of WP TCs are segregated into inner core (IC), inner rainbands (IB), and outer rainbands (OB) using the methods proposed in Yang et al.^20^.

The radar reflectivity profiles (for the period of 2005–2019) were used to generate the contour frequency by altitude diagrams (CFADs) over the disdrometer site (24.55° N–24.6° N, 121.0875° E–121.1375° E) are obtained from six ground-based radars’ (red color triangles in supplementary Fig. 1b) reflectivity mosaic, which is archived by central weather bureau of Taiwan at a spatial and temporal resolution of 0.0125° × 0.0125° and 10-min, respectively. A detailed description of six ground-based radars and the reflectivity mosaic can be found in Chang et al.^37^. Along with the disdrometer and radar reflectivity data sets, meteorological parameters from the NCU weather station, total column water vapor, total column ice water content, total column liquid water content, zero degrees isotherm, convective available potential energy values from the ERA5 are also used^38^.

## Supplementary Information

Supplementary Information.

## Data Availability

The datasets used and/or analysed during the current study available from the corresponding author on reasonable request.
