# Supplementary material for: An insight into the microphysical attributes of northwest Pacific tropical cyclones
Source: Sci Rep. 2023 Mar 17;13:4432. doi: 10.1038/s41598-023-29144-4 (PMC10023739; doi:10.1038/s41598-023-29144-4)
Supplement: Supplementary file 1 — Supplementary Information. [file 41598_2023_29144_MOESM1_ESM.docx]

Supplementary Information for

**An insight into the microphysical attributes of northwest Pacific tropical cyclones**

**
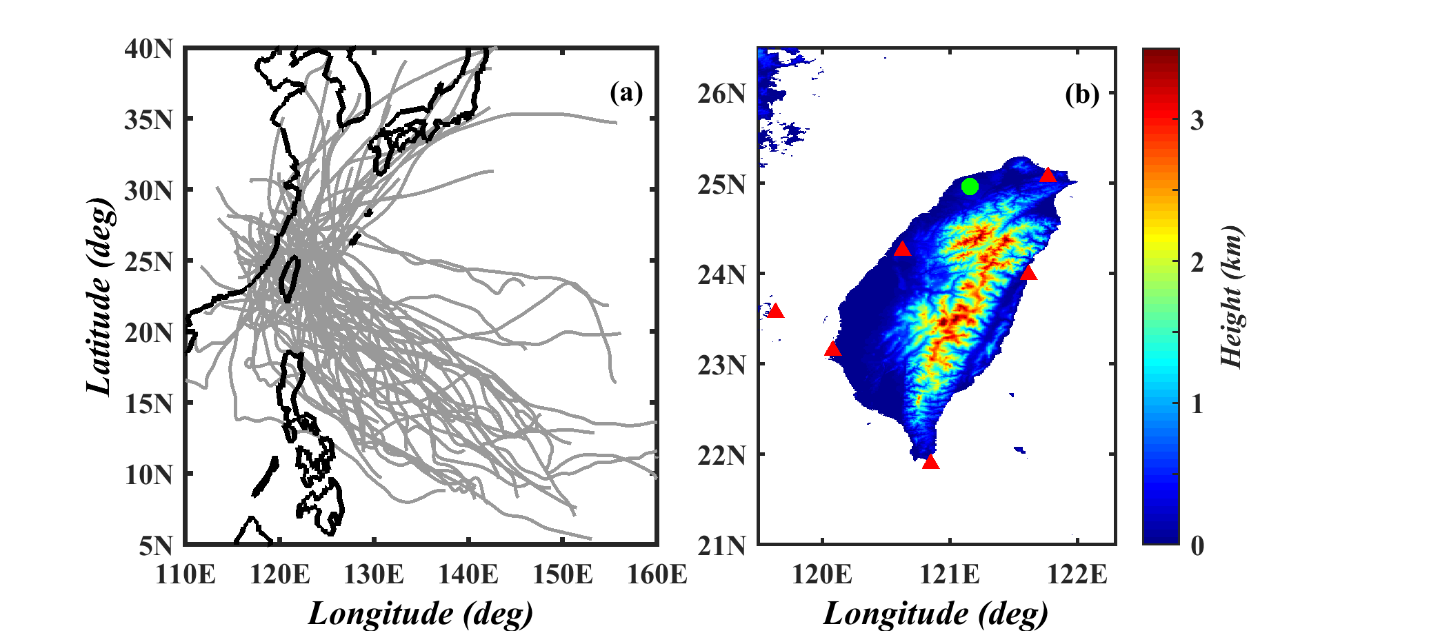
**

**Supplementary figure 1. (a)** The tracks of tropical cyclones considered in the present study **(b)** Taiwan terrain map with the location of disdrometer (green filled circle) and radars (red filled triangle).


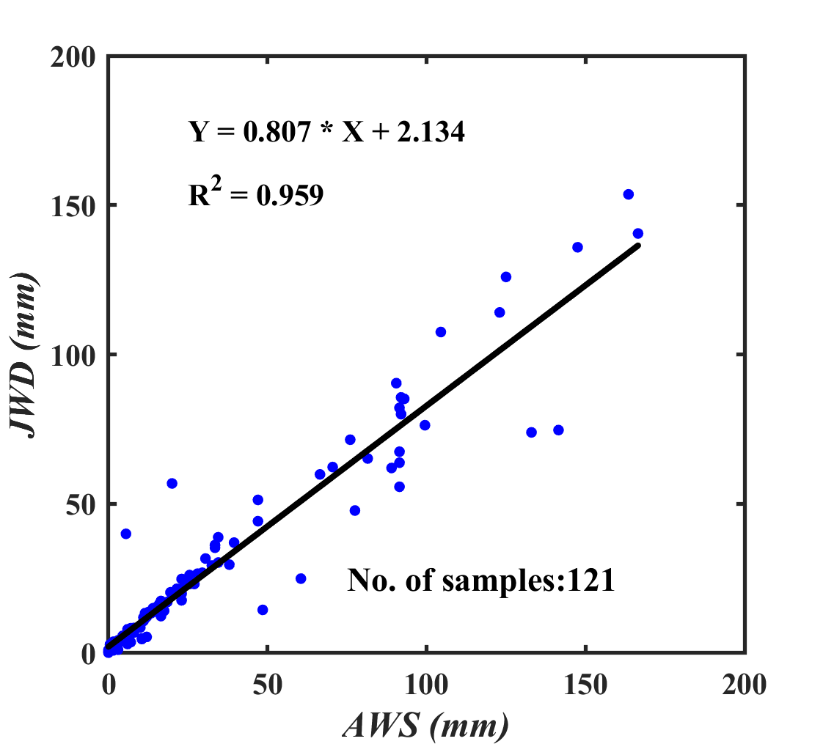


**Supplementary figure 2.** Scatter plot of disdrometer measured rainfall accumulations with collocated rain gauge.

The quality of disdrometer measurements are validated by comparing the daily rainfall accumulations of TCs from the disdrometer with the collocated rain gauge measurements. The comparison clearly a good agreement between disdrometer measurements with the rain gauge, which provides the confidence to utilized the disdrometer measurements for the further analysis.

**
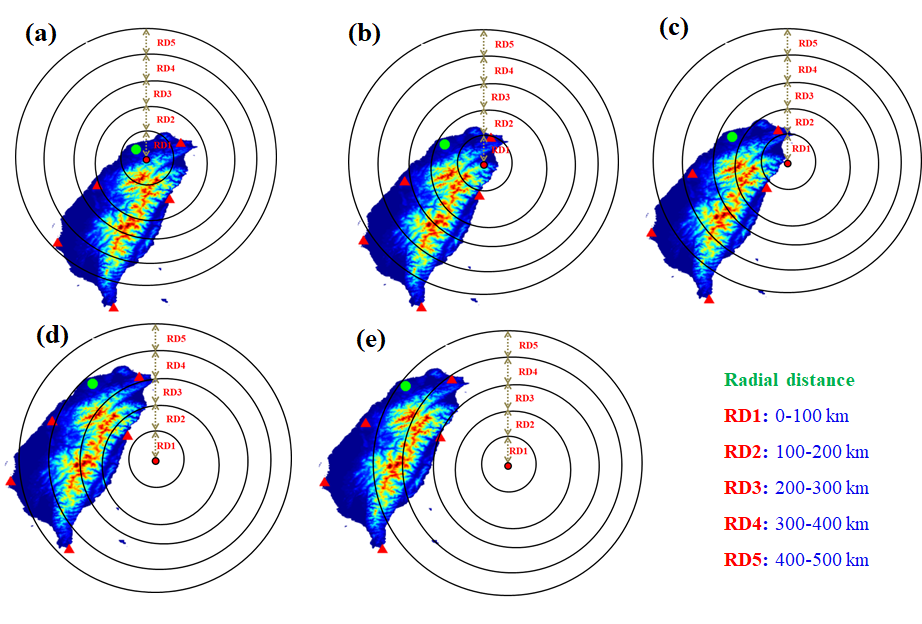
**

**Supplementary Figure 3** Schematic diagram of a tropical cyclone radial distances. Tropical cyclone RSD measurements by the disdrometer at (**a**) Radial distance 1 (RD1: 0–100 km), **(b)** radial distance 2 (RD2: 100–200 km), **(c)** radial distance 3 (RD3: 200–300 km), **(d)** radial distance 4 (RD4: 300–400 km), and **(e)** radial distance 5 (RD5: 400–500 km).


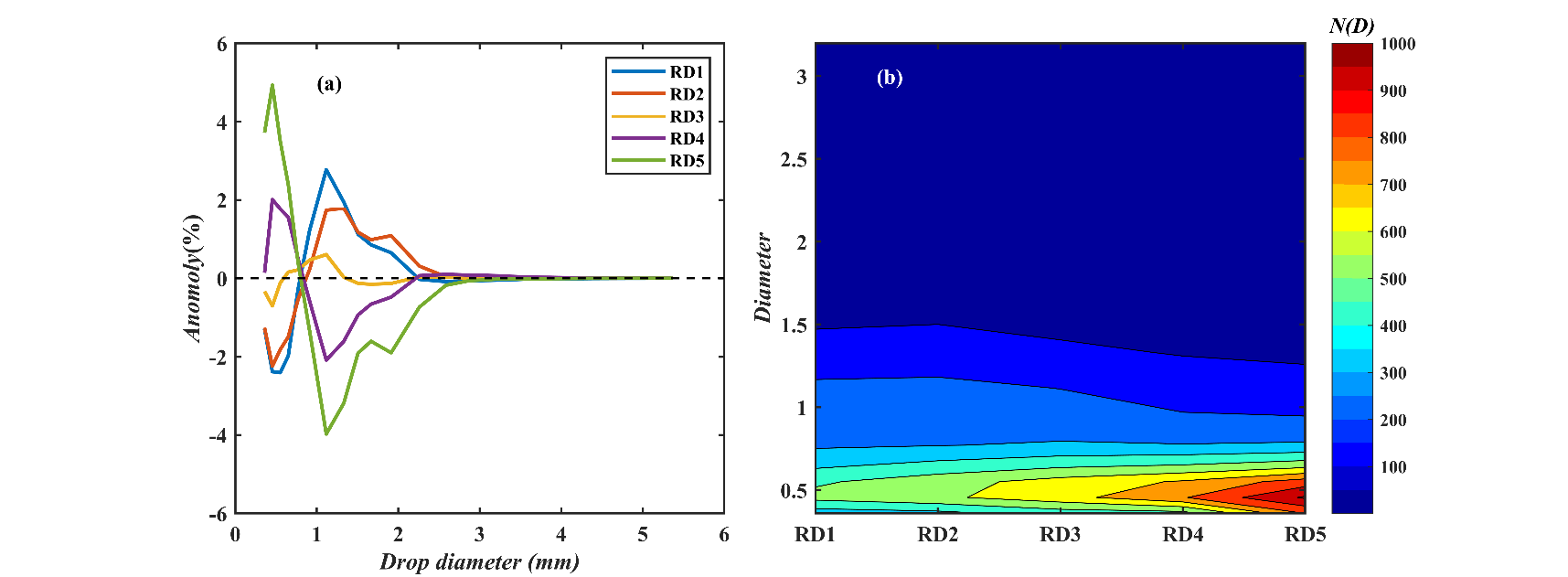


**Supplementary Figure 4 (a**) RSD frequency anomalies for different radial distances (RD1: 0–100 km, RD2: 100–200 km, RD3: 200–300 km, RD4: 300–400 km, and RD5: 400–500 km). (**b**) Contour plot of distribution of mean raindrop concentration (*N(D)*, m^–3^ mm^–1^) with TCs radial distance.

Supplementary Figure 4a displays the RSD anomaly at different radial distances (RD1: 0–100 km, RD2: 100–200 km, RD3: 200–300 km, RD4: 300–400 km, RD5: 400–500 km) from TCs centers. The Figure 4a demonstrates a positive anomaly in small drops (< 1mm) for the radial distances > 200 km and a negative anomaly for the radial distance < 200 km from TCs center. The contour frequency distribution of raindrop concentration (*N(D)*, m^–3^ mm^–1^) clearly establishes an increase in small drop concentration with the increase in radial distance from TCs centers.


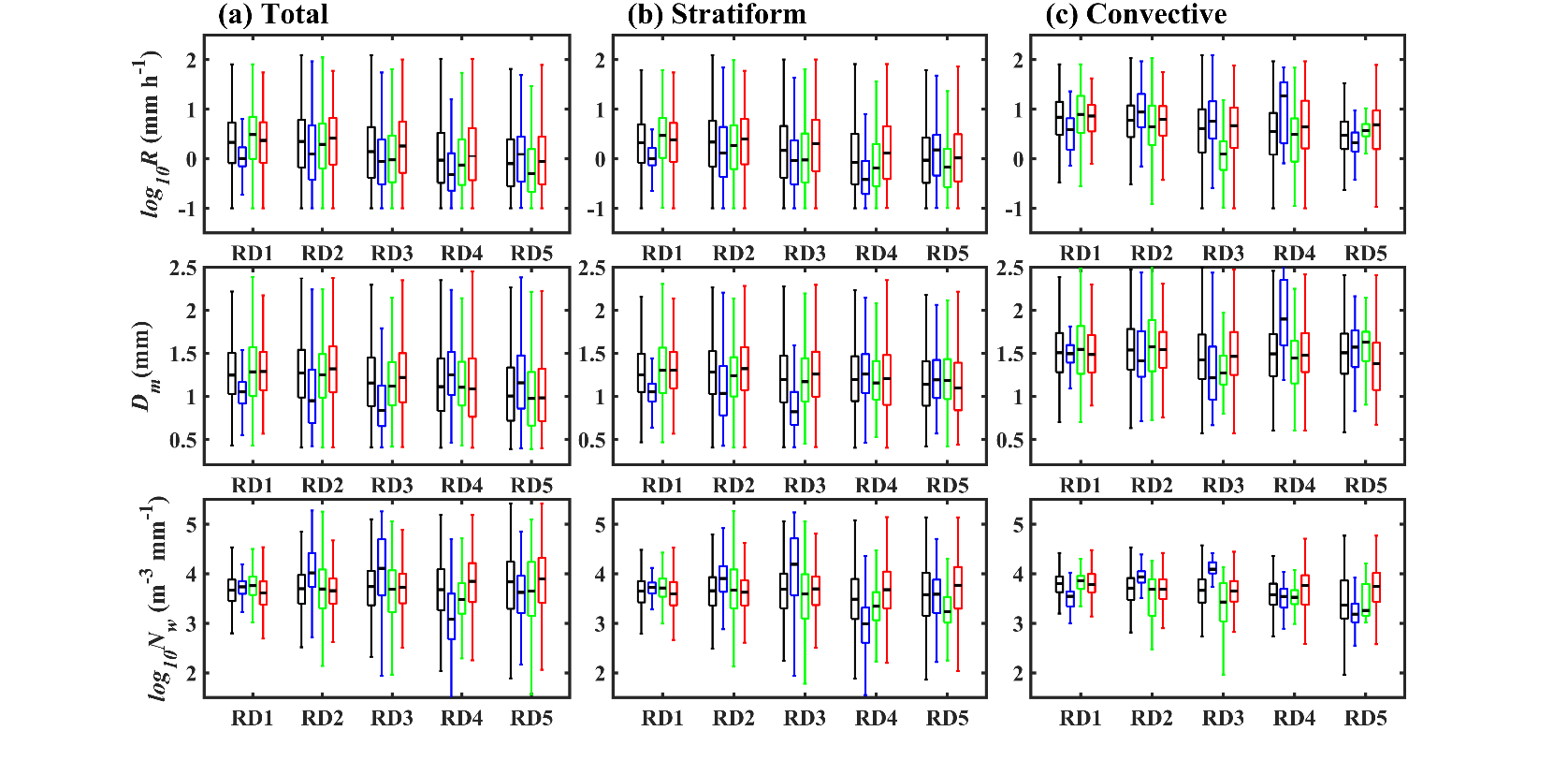


**Supplementary Figure 5** Distribution of **(a)** total, **(b)** stratiform, and **(c)** convective precipitation RSD parameters for TCs of different category and radial distances. Distribution of rainfall rate (log_10_*R*, *R* is in mm h^–1^), mass-weighted mean diameter (*D_m_*, mm), and normalized intercept parameter (log_10_*N_w_*, *N_w_* is in m^–3^ mm^–1^) for different radial distances (RD1: 0–100 km, RD2: 100–200 km, RD3: 200–300 km, RD4: 300–400 km, and RD5: 400–500 km) of all TCs (ALL: black color box plots), tropical depressions (TD: blue color box plots), tropical storms (TSs: green color box plots) and category1 to category 5 (CAT15: red color box plot).
